# A call for solutions-oriented research and policy to protect children from the effects of climate change

**DOI:** 10.1038/s41390-024-03559-9

**Published:** 2024-09-06

**Authors:** Patrick H. Ryan, Nicholas Newman, Kimberly Yolton, Jareen Meinzen-Derr, Tracy Glauser, Tina L. Cheng, on behalf of the Pediatric Policy Council, Shetal Shah, Mona Patel, Maya Ragavan, Scott Lorch, Lisa Chamberlain, Tina Cheng, Ann Reed, Joyce Javier, Ashwini Lakshmanan

**Affiliations:** 1 Department of Pediatrics, University of Cincinnati College of Medicine, Cincinnati, OH USA; 2 Division of Biostatistics and Epidemiology, Cincinnati Children’s Hospital Medical Center, Cincinnati, OH USA; 3 Division of General and Community Pediatrics, Cincinnati Children’s Hospital Medical Center, Cincinnati, OH USA; 4 Pediatric Policy Council, McLean, VA USA; 5 Academic Pediatric Association, McLean, VA USA; 6 American Pediatric Society, The Woodlands, TX USA; 7 Association of Medical School Pediatric Department Chairs, McLean, VA USA; 8 Society for Pediatric Research, McLean, VA USA

## Abstract

This month’s article “Exposure to Air Pollution and Cardiovascular Risk in Young Children – a Pilot Project” reports novel findings that young children exposed to low levels of air pollution have early indicators of vascular damage. These results add to our understanding of the wide-ranging health effects of air pollution exposure and serve as a reminder that children are highly vulnerable to environmental exposures. All children deserve healthy environments that promote their physical and mental development, but the consequences of climate change threaten this right. Extreme weather, heat, wildfires, flooding, infectious disease, and other climate change effects have wide-ranging impact on human health with children likely to bear the highest burden of disease due to climate change. The pediatric research community should meet the urgent need for solutions to this crisis by engaging in research that identifies effective strategies to address the health effects caused by current and future climate change to protect the health of children and future generations.

In this month’s issue, Groner and colleagues report novel data demonstrating that children as young as 2–5 years who are exposed to traffic-related air pollution (TRAP) have elevated levels of specific subtypes of endothelial progenitor cells (EPCs).^1^ These findings are notable for several reasons. First, prior studies of young children exposed to TRAP - a complex mixture of gases and particles emitted from motor vehicles – have identified adverse associations with birth outcomes,^2,3^ respiratory disease,^4–6^ and neurodevelopment,^7–9^ but its role as an early childhood risk factor for cardiovascular disease has not been thoroughly studied.^10^ Groner et al.’s findings, in combination with previous studies linking TRAP exposure among adults to increased cardiovascular disease (CVD), should encourage additional studies of environmental exposures and CVD risk among young children. Secondly, TRAP is a major contributor to outdoor air pollution, particularly in urban areas, and is elevated near major roadways.^11^ Exposure to TRAP disproportionately affects racial and ethnic minorities and residents of lower income neighborhoods despite these neighborhoods emitting proportionately less TRAP than more “advantaged” (and less polluted) neighborhoods.^12,13^ These groups also experience higher rates of CVD and other detrimental social determinants of health (SDoH) that amplify the impact of TRAP exposure.^14–17^ Third, and importantly, the concentrations of air pollutants in the Groner et al. report were lower than current regulatory limits established by the U.S. Environmental Protection Agency adding to the accumulating evidence that adverse health effects associated with air pollution exposure occur at levels below the current National Ambient Air Quality Standards.^18,19^

The results of Groner et al. are also a poignant reminder that children are profoundly shaped by the environments in which they grow up and the broader social, economic, and political contexts in which these environments exist. These influences, over which children have no control, have lifelong implications for physical and mental health and development. A healthy environment, beginning prenatally and continuing throughout childhood, is essential to physical and mental well-being across the lifespan. Children are especially susceptible to social and environmental determinants of health, both positive (e.g., access to healthy food, good air quality) and negative (e.g., lack of healthy food, poor air quality) as these can influence developing organs and physiological systems and alter the trajectory of physical and mental health and development.^20^

A broader appreciation for the role of healthy environments in children’s growth and development is urgently needed in the face of the accelerating climate change crisis.^21^ Since global records began in 1850, the ten warmest years on record have occurred in the past decade.^22^ This trend is continuing; as of this writing, June 2024 was the warmest June on record, surpassing the previous highest by 0.27 °F, a record set only the year prior. Indeed, the Intergovernmental Panel on Climate Change (IPCC) recently concluded that the risks of climate change are appearing faster and becoming more severe sooner than previously expected and that the window of opportunity to ‘secure a livable and sustainable future for all’ is rapidly closing.^23^ In 2019, Watts et al. urged health professionals to emphasize the ‘human face’ of climate change by connecting climate change to its health consequences. To this end, guidance to incorporate climate change discussions into pediatric primary care visits has been provided, and a recent policy statement by the American Academy of Pediatrics provides specific recommendations for pediatric clinicians to take a larger role in combating the effects of climate change on children.^24,25^

As a community of pediatric researchers, our work has unequivocally demonstrated the harmful effects of climate-related exposures on children’s health and the long-lasting implications of these consequences through adulthood. We know, through decades of research, that changing temperatures, extreme heatwaves, wildfires, hurricanes, and other downstream effects of climate change on air quality, food and water security, and infectious disease distribution will have immediate and long-term effects on child health and well-being.^21,25–29^ Multiple facets of childhood increase the impact of these exposures on children compared to adults including differences in physiology and metabolism, reduced immune function, higher exposures per unit of body weight, and varying time-activity patterns that may increase exposure (e.g., more time spent outdoors).^30^ We also know that the impact of these will be disproportionately experienced by low-income families, racial and ethnic minorities, and residents of low- and middle-income countries with the fewest resources to protect themselves and who contribute the least to greenhouse gasses.^21,31–33^

The urgency of the climate crisis requires us to engage in solutions-oriented research and identify effective strategies to address the health effects caused by current and future climate change. More than a decade ago, Xu et al. issued a call for research on climate-change and children’s health with a focus on identifying the most vulnerable subpopulations, projecting the burden of disease under varying climate change scenarios, and identifying cost-effective mitigation and adaption actions.^34^ While this is important work, we need to do more. We encourage researchers to reframe their thinking from identifying associations between climate-related exposures and health outcomes to studies of methods and approaches to protect children from the harm caused by climate change. The enormity of the problem requires systems-level thinking and solutions. Engaging with implementation scientists and team science partnerships with colleagues in urban planning and engineering to carry out evidence-based research and intervention strategies to mitigate and adapt to climate change is one approach. For example, urban green and blue spaces (land covered by vegetation and water, respectively) can help to combat climate-related heat waves and air pollution as these areas help to improve air quality, reduce urban heat islands, and encourage social connections and physical activity.^35–38^ Despite epidemiologic studies demonstrating improved mental, cognitive, behavioral, and physical health outcomes associated with green space, many lower income neighborhoods continue to be absent of these spaces. The disconnect between environmental health research knowledge and application of solutions demonstrates the importance of partnerships between implementation scientists, urban planners, and engineers to devise strategies that move from scientific reporting of associations to action targeting improved public health. New intervention studies should also be designed to test strategies at the individual level to lessen the health impacts of climate-related exposures. Determining the effectiveness of do-it-yourself air filters (e.g., Corsi-Rosenthal boxes) to improve indoor air quality is one concept that could be tested as a low-cost intervention for families affected by wildfire smoke exposure.^39^ At a policy level, researchers could partner with local governments and communities to identify the health implications of already identified plans to address the effects of climate change. These measures often include identifying the most climate vulnerable neighborhoods, expanding tree canopy, upgrading infrastructure, transitioning to electric vehicles, improving active transportation, and other local adaptation measures to mitigate the effects of climate-related exposures most relevant to their locales. We must also identify ways to reduce our own emissions given that health care facilities are a significant contributor to greenhouse gas emissions (approximately 8.5% of emissions) in the U.S.^40^

All children deserve to experience healthy built, social, and familial environments that promote their physical and mental health and development. Climate change is an existential threat to the health of every child that requires strategies aimed at both slowing the progression of and developing adaptations to our rapidly changing world. This work will require a concentrated multidisciplinary effort by environmental and health researchers, engineers, urban planners, policy experts, dissemination and implementation scientists, and government agencies. The strategic framework of the NIH Climate Change and Health Initiative program identifies health effects research, health equity, intervention research, training, and capacity building as key areas of interest and calls for solution-focused research and intervention science to ‘develop targeted preventions and adaptations’.^41^ We urge pediatric researchers to consider answering this call to ensure a sustainable future and safeguard the health and well-being of children and future generations.
