# Correction: a call for solutions-oriented research and policy to protect children from the effects of climate change

**DOI:** 10.1038/s41390-024-03593-7

**Published:** 2024-09-25

**Authors:** Patrick H. Ryan, Nicholas Newman, Kimberly Yolton, Jareen Meinzen-Derr, Tracy Glauser, Tina L. Cheng, on behalf of the Pediatric Policy Council, Shetal Shah, Mona Patel, Maya Ragavan, Scott Lorch, Lisa Chamberlain, Tina Cheng, Ann Reed, Joyce Javier, Ashwini Lakshmanan

**Affiliations:** 1 Department of Pediatrics, University of Cincinnati College of Medicine, Cincinnati, OH USA; 2 Division of Biostatistics and Epidemiology, Cincinnati Children’s Hospital Medical Center, Cincinnati, OH USA; 3 Division of General and Community Pediatrics, Cincinnati Children’s Hospital Medical Center, Cincinnati, OH USA; 4 Pediatric Policy Council, McLean, VA USA; 5 Academic Pediatric Association, McLean, VA USA; 6 American Pediatric Society, The Woodlands, TX USA; 7 Association of Medical School Pediatric Department Chairs, McLean, VA USA; 8 Society for Pediatric Research, McLean, VA USA

Correction to: *Pediatric Research* 10.1038/s41390-024-03559-9, published online 06 September 2024

In the original article, the ‘Pediatric Policy Council’ was inadvertently omitted from the author list. The author list should read: “Patrick H. Ryan^1,2^, Nicholas Newman^1,3^, Kimberly Yolton^1,3^, Jareen Meinzen-Derr^1,2^, Tracy Glauser^1^ and Tina L. Cheng^1,*^ on behalf of the Pediatric Policy Council.” The members of the Pediatric Policy Council have been added at the end of the article: “Shetal Shah (Chair), Mona Patel (Academic Pediatric Association), Maya Ragavan (Academic Pediatric Association), Scott Lorch (American Pediatric Society), Lisa Chamberlain (American Pediatric Society), Tina Cheng (Association of Medical School Pediatric Department Chairs), Ann Reed (Association of Medical School Pediatric Department Chairs), Joyce Javier (Society for Pediatric Research) & Ashwini Lakshmanan (Society for Pediatric Research).” The original article has been corrected.

